# Oral Esketamine Maintenance Treatment for Treatment‐Resistant Depression: Clinical Outcomes During an Off‐Label Treatment Program

**DOI:** 10.1155/da/8320239

**Published:** 2026-10-29

**Authors:** Sanne Y. Smith-Apeldoorn, Jolien K. E. Veraart, Jan Spijker, Annemarie Van Der Meij, Jeanine Kamphuis, Robert A. Schoevers

**Affiliations:** ^1^ Department of Psychiatry University of Groningen University Medical Center Groningen Groningen Netherlands umcg.nl; ^2^ Department of Psychiatry PsyQ Haaglanden Parnassia Psychiatric Institute The Hague Netherlands; ^3^ Depression Expertise Center Pro Persona Mental Health Care Nijmegen Netherlands; ^4^ Behavioral Science Institute Radboud University Nijmegen Netherlands ru.nl

## Abstract

Ketamine and esketamine maintenance treatment show promise in maintaining initial antidepressant effects, yet further research is needed to optimize long‐term outcomes. This observational study aimed to assess long‐term depression symptom severity, safety, and tolerability in a cohort of patients receiving at‐home oral esketamine maintenance treatment. Between June 2017 and March 2024, 221 patients with severe treatment‐resistant unipolar depression were enrolled directly or trial‐transferred into an off‐label induction treatment program. After 6 weeks of individually titrated oral esketamine (twice a week, max 3.0 mg/kg) administered in the clinic, 93 patients continued to maintenance treatment at home (max 3.0 mg/kg twice a week). Our primary outcome was change in depression symptom severity during maintenance treatment, measured by the 17‐item Hamilton Depression Rating Scale (HDRS_17_). Mean exposure to esketamine in the maintenance phase was 20.8 months. We found no difference between the HDRS_17_ total score at the maintenance baseline and last‐observation‐carried‐forward (mean difference = 0.825, 95% CI = −0.759 to 2.410, *p* = 0.302). Of the 26 initial responders (≥50% decrease in HDRS_17_ at the end of induction treatment), 23.1% met sustained response criteria throughout maintenance treatment. Of the 40 patients meeting minimal clinically important difference (MCID) criteria (≥27.1% decrease in HDRS_17_ at the end of induction treatment), 60.0% met sustained MCID criteria throughout maintenance treatment. Eleven patients (11.8%) successfully tapered off esketamine maintenance treatment. Twelve patients (12.9%) discontinued maintenance treatment (partly) due to adverse events. These findings suggest that oral esketamine maintenance home treatment may be associated with sustained symptom stability in patients with severe treatment‐resistant depression (TRD), although causal inferences cannot be drawn from this observational study. Although some potential long‐term side effects require monitoring and further examination, overall, treatment was safe and well‐tolerated.

## 1. Introduction

Rapid and robust reductions in symptoms of depression have been shown following the administration of ketamine and its enantiomer, esketamine, including in patients with treatment‐resistant depression (TRD) [1, 2]. However, these effects are often short‐lived. Relapse rates are high after cessation of treatment, and the median time to relapse is only a few weeks [3–6]. Several non‐ketamine strategies have been explored over the past decade to prolong the initial benefits of ketamine and esketamine, unfortunately, with limited success so far [7–9]. Therefore, ketamine or esketamine maintenance treatment may be necessary strategy to sustain their initial antidepressant effects.

The most recent studies and a systematic review by our team in 2022 showed the substantial therapeutic potential of ketamine and esketamine maintenance treatment for depression within the first 6 months. Results of maintenance treatment beyond 6 months are more scarce but appear promising nonetheless [10–13]. Potentially serious safety concerns of long‐term ketamine and esketamine treatment have been posited following observations of cognitive impairment, renal and urinary tract problems, hepatotoxicity, and dependence with prolonged or frequent recreational use of ketamine [14–17]. These appear to be uncommon in ketamine and esketamine maintenance treatment for depression [10, 11, 18]. However, results regarding both sustained antidepressant effects and safety should be interpreted with caution, as the overall quality of the majority of the maintenance studies is considered low to moderate, with most reports showing a high risk of bias in several domains [10, 19]. Therefore, both controlled and naturalistic ketamine and esketamine maintenance studies with long‐term follow‐up and sufficient statistical power are needed to establish their real‐world potential [20].

Ketamine and esketamine can be administered in various ways. While intravenous (IV) ketamine is recognized as the most robustly supported route of administration in the literature, the repeated use of IV infusions presents limited feasibility, accessibility, and scalability. The efficacy of intranasal (IN) esketamine for TRD has been shown in both short‐term and longer‐term studies [11, 21]. However, accessibility and scalability of the nasal spray are also limited as patients are required to visit the clinic for each treatment, as well as due to its costs [22, 23]. At its current pricing, the nasal spray is considered unlikely to be cost‐effective for the management of TRD [24]. The subcutaneous (SC) and intramuscular (IM) routes appear relatively simple, safe, and effective but require further testing [25–27].

Most regular antidepressant medications are administered orally, which is the preferred route of administration by patients [28] and offers the greatest potential for accessibility and scalability. Positive results of induction treatment with oral ketamine and esketamine have been found in small and uncontrolled studies and a few randomized controlled trials [29–31]. The results of a randomized placebo‐controlled trial by our team suggest that daily, fixed, low‐dose oral esketamine is not effective for TRD [32]. In contrast, based on the results of the ongoing open‐label treatment program we initiated in 2017, individually titrated higher doses of oral esketamine administered twice a week appear to have antidepressant properties [32, 33].

After 6 weeks of induction treatment, 47.1% of our patients with severe TRD showed clinically relevant improvement [33]. Although these results should be interpreted with caution due to the limitations of the study design, oral esketamine clearly appears to have antidepressant properties.

After the 6‐week induction treatment with flexible‐dose oral esketamine administered in the clinic [32, 33], patients perceiving clinically relevant improvement are offered at‐home oral esketamine maintenance treatment. As with other applications, data on oral ketamine and esketamine maintenance treatment are scarce [10], emphasizing the importance of further research. Therefore, in this article, we assess long‐term depression symptom severity, safety, and tolerability in a cohort of patients receiving at‐home oral esketamine maintenance treatment.

## 2. Methods

### 2.1. Study Design

This study involved a cohort of patients with severe TRD. Patients enrolled in an ongoing oral esketamine treatment program at three psychiatric institutes in the Netherlands—the University Medical Center Groningen (UMCG), Pro Persona Mental Health Care in Nijmegen, and Parnassia Psychiatric Institute in The Hague—between June 2017 and March 2024 were included. This treatment program consists of two phases: (1) a 6‐week off‐label induction treatment during which patients receive individually titrated oral esketamine twice a week at clinical sites and (2) an optional off‐label maintenance treatment for an undetermined period of time during which patients receive individually titrated oral esketamine at home, with a maximum of two times a week. The treatment program and outcome measurement protocol were approved by the Medical Ethics Review Committee of the UMCG in the Netherlands as not subject to the Medical Research Involving Human Subjects Act (File Number 2017/446). Results of the 6‐week induction treatment have been previously described [32, 33]. In this article, we report outcomes observed during the maintenance treatment program based on a database lock on March 27th, 2024.

### 2.2. Patients

Inclusion and exclusion criteria for the off‐label induction treatment program have been described in detail elsewhere [33]. In summary, patients were eligible if they (1) had participated in the randomized placebo‐controlled trial on fixed low‐dose oral esketamine [32, 34] (transferred entry) or (2) were aged 18 years or older, had a current depressive episode according to the Diagnostic and Statistical Manual of Mental Disorders fifth edition (DSM‐5) as determined by the Mini International Neuropsychiatric Interview (MINI) [35], and met the off‐label treatment program definition of TRD, indicating that the patient could not be treated satisfactorily with any of the conventional treatment steps according to the Dutch guideline for treatment of depression (direct‐entry) [36, 37]. TRD was defined according to the compassionate use recommendations of the European Medicines Agency (EMA): products in development can be made available to patients who have a long‐lasting and seriously debilitating disease with no satisfactory authorized therapies and who cannot enter clinical trials [38]. The main exclusion criteria were the inability to provide informed consent, a moderate to severe active substance use disorder (excluding tobacco), use of benzodiazepines in excess of 2 mg lorazepam or an equivalent per day, a somatic contraindication according to the esketamine summary of product characteristics (SmPC) [39], and pregnancy or lactation. A lifetime history of bipolar disorder was not an exclusion criterion, but outcomes observed in patients with bipolar depression will be published separately.

In the first 2 years of the program (until September 2019, *N* = 22), the decision to continue oral esketamine treatment after the 6‐week induction treatment was made through a moral case deliberation, given the limited empirical evidence and clinical experience with maintenance ketamine or esketamine treatment at that time. Participants in these moral case deliberations were the patient, a relative or carer, the referring physician, the physician providing esketamine treatment, and initially also an ethicist and members of the esketamine research team. In September 2019, this moral case deliberation was replaced by a regular shared decision‐making evaluation process, as is customary in clinical practice.

Patients were eligible for maintenance treatment if (1) clinically relevant improvement in depressive symptoms or quality of life was perceived by the patient at the end of the induction phase (i.e., this involved a qualitative assessment; no quantified definition was determined given the last‐resort status of the treatment for the eligible patients—see criterion 3), (2) oral esketamine’s side effects were acceptable to both the patient and physician based on clinical judgment and in shared decision‐making, and (3) there were no remaining conventional treatment options according to the Dutch guideline for treatment of depression [36, 37]. All patients enrolled in the current cohort provided written informed consent for off‐label treatment and for the collection and publication of data.

### 2.3. Treatment Procedure

The 6‐week induction treatment has been described in detail elsewhere [33]. In summary, patients received 12 doses of esketamine (10 mg/mL, liquid formulation) administered at one of the three participating clinics twice a week over a 6‐week period. Esketamine was initiated on day 1 at 0.5 or 1.0 mg/kg, with the possibility, per physician’s clinical judgment based on effectiveness and tolerability, of decreasing or increasing the dose to a maximum of 2.0 mg/kg (June 2017–April 2019) or 3.0 mg/kg (May 2019–March 2024). This increase in the maximum dose was based on a growing understanding of the significant interindividual differences in bioavailability of oral esketamine [33]. Patients continued their prescribed psychiatric medications and psychological treatment.

In the case of maintenance treatment after the induction treatment, oral esketamine was continued at home. Patients obtained a supply of esketamine from the clinic or the pharmacy every 2 weeks, prescribed by their local physician or a physician from one of the participating psychiatric institutes. At the start of the maintenance treatment, the dosage regimen (i.e., dose and frequency) remained unchanged from the final dosage regimen of the induction treatment. However, in the case of depression relapse perceived by the patient (i.e., this involved a qualitative assessment of the patient’s experience), dosing could be increased to the aforementioned maximum of two times a week, per the physician’s clinical judgment based on symptom trajectories and tolerability. In addition, patients and their physicians providing esketamine treatment were advised to consider (1) gradually decreasing the esketamine treatment frequency on a trial basis within 6 months because of the limited empirical evidence for maintenance esketamine treatment in depression, (2) discontinue esketamine treatment in the case of unacceptable side effects or risks, and (3) discontinue esketamine treatment in the case of depression relapse despite dosing increase to the aforementioned maximum of two times a week.

### 2.4. Outcome Measures

As part of the clinical maintenance treatment protocol, the 17‐item Hamilton Depression Rating Scale (HDRS_17_) [40] was administered by trained monitoring clinicians, either by phone or in the clinic, once a month to all patients until January 2021. From January 2021, the HDRS_17_ was administered once a month during the first 6 months of maintenance treatment, once every 3 months after 6 months of maintenance treatment, and once every 6 months after 1 year of maintenance treatment. In addition, the Inventory of Depressive Symptomatology—Self Report (IDS‐SR) [41] and Systematic Assessment for Treatment Emergent Events (SAFTEE) [42] were sent to all patients by email once a month throughout the maintenance treatment. Furthermore, physicians providing esketamine treatment were advised to test liver enzyme levels, kidney function, and cognitive function every 6 months. These measures are not included in this study. In the case of discontinuation of maintenance treatment, the reason for discontinuation (i.e., “insufficient (sustained) improvement,” and/or “adverse events,” and/or “other” with further details) and the duration of maintenance treatment were documented.

Our primary outcome was the trajectory of depressive symptom severity during oral esketamine maintenance treatment, measured by (1) change in depression symptom severity, expressed as a change in total HDRS_17_ score (primary outcome measure) and total IDS‐SR score from the maintenance baseline (i.e., the end of the induction treatment) to the last‐observation‐carried‐forward, irrespective of the duration of maintenance treatment, (2) sustained response, defined as the preservation of ≥ 50% decrease in HDRS_17_ total score from the baseline of the induction treatment to the end of the induction treatment (i.e., initial response) throughout maintenance treatment (i.e., sustained response), and (3) sustained minimal clinically important difference (MCID), defined as the preservation of ≥ 27.1% decrease in HDRS_17_ total score [43] from the baseline of the induction treatment to the end of the induction treatment (i.e., initial MCID) throughout maintenance treatment (i.e., sustained MCID).

General adverse events were recorded using the SAFTEE, a questionnaire that rates the severity of a wide range of symptoms, regardless of whether or not they are suspected to be drug‐related. The data provided depict how many patients rated a symptom as more bothersome at any point during maintenance treatment compared to the baseline rating. A moderate increase was defined as an increase of two degrees of severity (i.e., from “not present” to “moderate” or from “mild” to “severe”) and a severe increase as an increase of three degrees of severity (i.e., from “not present” to “severe”).

The tolerability of at‐home oral esketamine maintenance treatment was expressed by the discontinuation of treatment due to adverse events. For the tolerability profile, only adverse events evaluated as suspected adverse drug reactions per the physician’s clinical judgment were taken into account.

### 2.5. Statistical Analysis

Statistical analyses were performed using the IBM SPSS Statistics, version 28.0. HDRS_17_ and IDS‐SR data were analyzed using paired sample *t*‐tests, comparing severity scores between baseline and last‐observation‐carried‐forward. For all statistical tests, assumptions were checked, tests were two‐tailed, and results were considered significant at a *p*‐value of 0.05. Sustained response and MCID rates and reasons for discontinuation were summarized descriptively based on all available data. SAFTEE data were also summarized descriptively; however, due to the large volume of data, a subset of data was created, including only 6‐monthly measurements (± 1 month in case of missing data).

## 3. Results

### 3.1. Patients

Between June 2017 and March 2024, 221 patients were enrolled in the 6‐week induction phase. Of these, 105 (47.5%) entered the maintenance treatment program, of whom 93 were patients with unipolar depression. At the time of the database lock, 38 of these 93 patients (40.9%) were still receiving maintenance treatment. Two patients (2.2%) were lost to follow‐up, and 53 patients (57.0%) had discontinued esketamine maintenance treatment at some point, including 11 (11.8%) after successful tapering (Figure 1). Patients’ baseline characteristics are summarized in Table 1.

**Figure 1:**
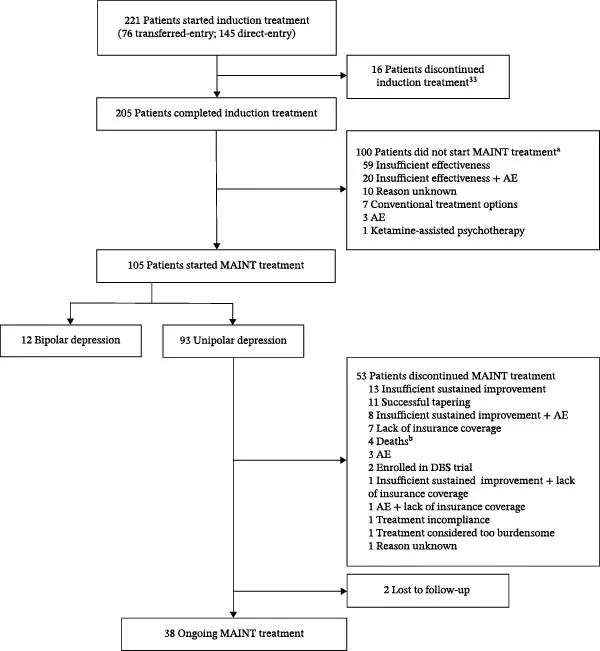
Flow diagram. ^a^For clinical characteristics of the patients who did not enter the maintenance treatment program see Table S1. ^b^Three patients died by physician‐assisted death, one patient died from pneumonia. AE = adverse events; DBS = deep brain stimulation; MAINT = maintenance.

**Table 1:** Baseline demographic and clinical characteristics of patients with a treatment‐resistant unipolar depressive disorder treated with oral esketamine maintenance treatment.

Characteristic	Maintenance treatment *N* = 93
Sex	
Male, *N* (%)	27 (29.0)
Female, *N* (%)	66 (71.0)
Age at baseline, years, mean (SD)	47.9 (13.91)
Age at onset, years, mean (SD)^a^	25.0 (12.87)
Duration current episode at baseline, months, mean (SD)^a^	79.9 (85.46)
No. of depressive episodes, mean (SD)^a^	8.9 (19.82)
No. of antidepressants lifetime, mean (SD)^b^	8.1 (3.93)
Antidepressant treatment at maintenance baseline^c^	
SSRI, *N* (%)	18 (22.2)
SNRI, *N* (%)	13 (16.0)
TCA, *N* (%)	24 (29.6)
Lithium (monotherapy), *N* (%)	2 (2.5)
Augmentation therapy, *N* (%)^d^	28 (34.6)
MAOi, *N* (%)	9 (11.1)
Other, *N* (%)^e^	33 (40.7)
History of ECT, *N* (%)^c^	53 (65.4)
No. of psychotherapies lifetime, mean (SD)^b^	4.9 (10.94)
Current psychotherapy treatment, *N* (%)^c^	38 (46.9)
Comorbid axis I disorder, *N* (%)^c^	60 (74.1)

Abbreviations: ECT, electroconvulsive therapy; MAOi, monoamine oxidase inhibitor; SNRI, selective serotonin and norepinephrine reuptake inhibitor; SSRI, selective serotonin reuptake inhibitor; TCA, tricyclic antidepressant.

^a^ *N* = 90.

^b^ *N* = 80.

^c^ *N* = 81.

^d^For example lithium, antipsychotic medication, triiodothyronine, stimulant medication.

^e^For example mirtazapine, bupropion, agomelatine, vortioxetine, mianserin.

### 3.2. Treatment Characteristics

At the time of the database lock, mean exposure to esketamine maintenance treatment was 20.8 months (SD = 18.18, range = 1–80; data regarding treatment duration are available for *N* = 90). For patients still receiving esketamine, the mean exposure was 31.1 months (SD = 15.94, range = 1–65, *N* = 38). Patients who had discontinued maintenance treatment after successful tapering had a mean exposure of 18.8 months (SD = 21.10, range = 3–80, and *N* = 11). Patients who had discontinued maintenance treatment for other reasons had a mean exposure of 11.7 months (SD = 14.21, range = 1–64; data regarding treatment duration are available for *N* = 41).

### 3.3. Symptom Severity

Figure 2 shows the mean HDRS_17_ total score over time. A paired sample *t*‐test including 63 patients (i.e., all patients with an available maintenance baseline and at least one available follow‐up HDRS_17_ total score) showed no significant change between baseline HDRS_17_ (mean = 12.63, SD = 6.524) and last‐observation‐carried‐forward HDRS_17_ (mean = 13.46, SD = 7.828) (mean difference = 0.825, SD = 6.293, *t* = 1.041, df = 62, *p* = 0.302, 95% CI = −0.759 to 2.410), indicating stability in depressive symptom severity at the group level during maintenance treatment.

**Figure 2:**
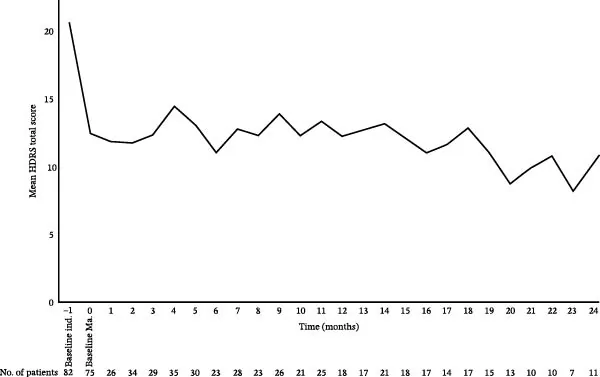
Mean Hamilton Depression Rating Scale total score. Mean Hamilton Depression Rating Scale (HDRS_17_) total score at baseline of the induction phase (T‐1: mean = 20.59, SE = 0.71, *N* = 82), at baseline of the maintenance phase (T0: mean = 12.43, SE = 0.73, *N* = 75), and over the course of maintenance treatment up to 24 months. For more details see Table S2. Severity interpretation of HDRS_17_ total scores: 0–7 = none (no depression); 8–13 = mild; 14–18 = moderate; 19–22 = severe; ≥23 = very severe.

A paired sample *t*‐test in the subgroup of patients still receiving maintenance treatment at the time of the database lock (data available for 28 of 38 patients) and in the subgroup of patients who discontinued maintenance treatment (partly) due to insufficient sustained improvement (data available for 16 of 22 (See Figure 1: 13 + 8 + 1) patients) showed similar results. In the first subgroup, the change in the HDRS_17_ total score from baseline (mean = 10.75, SD = 4.394) to the last observation (mean = 12.36, SD = 7.479) was not statistically significant (mean difference = 1.607, SD = 6.367, *t* = 1.336, df = 27, *p* = 0.193, 95% CI = −0.862 to 4.076). Also, in the second subgroup, the change in HDRS_17_ total score from baseline (mean = 17.06, SD = 8.354) to last observation (mean = 18.25, SD = 7.019) was not statistically significant (mean difference = 1.188, SD = 6.504, *t* = 0.730, df = 15, *p* = 0.476, 95% CI = −2.278 to 4.653). These data indicate that, on average, patients who were still receiving maintenance treatment had mild depressive symptoms [44] at both the maintenance baseline and the last observation, while patients who discontinued maintenance treatment (partly) due to insufficient sustained improvement had moderate depressive symptoms [44] at both the maintenance baseline and the last observation.

Stability in depressive symptom severity at the group level during maintenance treatment was supported by the IDS‐SR data. Figure 3 shows the mean IDS‐SR total score over time. A paired sample *t*‐test including 64 patients (i.e., all patients with an available maintenance baseline and at least one available follow‐up IDS‐SR total score) showed no significant change between baseline IDS‐SR (mean = 33.59, SD = 14.155) and last‐observation‐carried‐forward IDS‐SR (mean = 35.42, SD = 15.363) (mean difference = 1.828, SD = 10.384, *t* = 1.408, df = 63, *p* = 0.164, 95% CI = −0.766 to 4.422).

**Figure 3:**
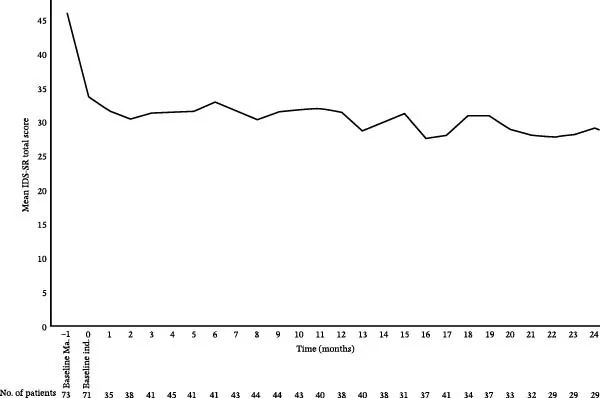
Mean Inventory of Depressive Symptomatology—Self Report total score. Mean Inventory of Depressive Symptomatology—Self Report (IDS‐SR) total score at baseline of the induction phase (T‐1: mean = 46.08, SE = 1.40, *N* = 73), at baseline of the maintenance phase (T0: mean = 33.73, SE = 1.72, *N* = 71), and over the course of maintenance treatment up to 24 months. For more details see Table S3. Severity interpretation of IDS‐SR total scores: 0–13 = none (no depression); 14–25 = mild; 26–38 = moderate; 39–48 = severe; ≥49 = very severe.

In the subgroup of patients still receiving maintenance treatment at the time of the database lock (data available for 25 of 38 patients), change in the IDS‐SR total score from baseline (mean = 29.88, SD = 12.647) to the last observation (mean = 31.76, SD = 13.264) was not statistically significant (mean difference = 1.880, SD = 10.772, *t* = 0.873, df = 24, *p* = 0.391, 95% CI = −2.566 to 6.326). Similarly, in the subgroup of patients who discontinued maintenance treatment (partly) due to insufficient sustained improvement (data available for 16 of 22 patients), change in the IDS‐SR total score from baseline (mean = 42.69, SD = 12.098) to the last observation (mean = 45.75, SD = 12.157) was not statistically significant (mean difference = 3.063, SD = 8.536, *t* = 1.435, df = 15, *p* = 0.172, 95% CI = −1.486 to 7.611). These data indicate that, on average, patients who were still receiving maintenance treatment had moderate depressive symptoms [44] at both maintenance baseline and the last observation, while patients who discontinued maintenance treatment (partly) due to insufficient sustained improvement had severe depressive symptoms [44] at both maintenance baseline and the last observation.

Data for the examination of response and MCID (i.e., HDRS_17_ assessment before and at the end of the induction phase to determine the initial response and MCID and at least one HDRS_17_ assessment during maintenance treatment to determine the sustained response and MCID) were available for 61 patients. At the end of induction treatment (i.e., baseline of maintenance treatment), 40 patients (65.6% of *N* = 61) had met initial MCID criteria, of whom 26 patients (42.6% of *N* = 61) met initial response criteria. Next, examination of the available individual‐level follow‐up data revealed that six of the 26 responders (23.1%; 9.8% of *N* = 61) met sustained response criteria (mean treatment duration 29.0 months (SD = 12.02)). Of the 40 patients who met initial MCID criteria, 24 (60.0%; 39.3% of *N* = 61) met sustained MCID criteria (mean treatment duration 22.6 months (SD = 18.45)).

Further exploration of the individual‐level data showed several distinct trajectories. Of the 26 initial responders, 14 (53.8%) met response criteria intermittently, and six (23.1%) (partly) relapsed and did not meet response criteria again during maintenance treatment (mean time to (first) relapse 6.5 months (SD = 4.85)). This was offset by 17 (48.6%) of the 35 initial nonresponders who met response criteria after months of maintenance treatment, of which eight (47.1% of *N* = 17 and 22.9% of *N* = 35) maintained response throughout the remainder of the treatment. Of the 40 patients who met initial MCID criteria, nine (22.5%) met MCID criteria intermittently, and seven (17.5%) (partly) relapsed and did not meet MCID criteria again during maintenance treatment (mean time to (first) relapse 6.7 months (SD = 5.33)). Fourteen (66.7%) of the 21 patients who did not meet MCID criteria at the end of induction treatment met MCID criteria after months of maintenance treatment, of which six (42.9% of *N* = 14 and 28.6% of *N* = 21) met MCID criteria throughout the remainder of maintenance treatment.

### 3.4. Safety and Tolerability

Maintenance baseline SAFTEE data and data from at least one follow‐up SAFTEE were available for 60 patients (Table 2). A moderate increase in symptoms at any time during maintenance treatment was reported 239 times, with the most common symptoms being excessive sweating (20.0%), nightmares or other sleep disturbance (16.7%), poor memory (13.3%), weakness or fatigue (13.3%), and increased appetite (13.3%). A severe increase in symptoms at any time during the maintenance treatment was reported 68 times, with the three most common symptoms being delayed or absent orgasm (11.7%), problems with sexual arousal (8.3%), and trouble sleeping (8.3%).

**Table 2:** Moderate‐to‐severe increase in symptoms on the Systematic Assessment for Treatment Emergent Effects throughout oral esketamine maintenance treatment.

Item	Moderate increase	Severe increase
Delayed or absent orgasm, *n* (%)	3 (5.0%)	7 (11.7%)
Problems with sexual arousal, *n* (%)	6 (10.0%)	5 (8.3%)
Trouble sleeping, *n* (%)	6 (10.0%)	5 (8.3%)
Loss of sexual interest, *n* (%)	4 (6.7%)	4 (6.7%)
Decreased appetite, *n* (%)	7 (11.7%)	3 (5.0%)
Dry mouth, *n* (%)	6 (10.0%)	3 (5.0%)
Apathy, *n* (%)	3 (5.0%)	3 (5.0%)
Menstrual irregularities, *n* (%)	2 (3.3%)	3 (5.0%)
Sweating excessively, *n* (%)	12 (20%)	2 (3.3%)
Nightmares or other sleep disturbance, *n* (%)	10 (16.7%)	2 (3.3%)
Poor memory, *n* (%)	8 (13.3%)	2 (3.3%)
Diminished mental capacity, *n* (%)	7 (11.7%)	2 (3.3%)
Nausea or vomiting, *n* (%)	6 (10.0%)	2 (3.3%)
Skin rash or allergy, *n* (%)	4 (6.7%)	2 (3.3%)
Headache, *n* (%)	3 (5.0%)	2 (3.3%)
Hearing or seeing things, *n* (%)	2 (3.3%)	2 (3.3%)
Hot flashes, *n* (%)	2 (3.3%)	2 (3.3%)
Stuffy nose, *n* (%)	0 (0.0%)	2 (3.3%)
Numbness or tingling, *n* (%)	0 (0.0%)	2 (3.3%)
Irritable, *n* (%)	7 (11.7%)	1 (1.7%)
Stomach or abdominal discomfort, *n* (%)	7 (11.7%)	1 (1.7%)
Weight gain, *n* (%)	7 (11.7%)	1 (1.7%)
Weight loss, *n* (%)	6 (10.0%)	1 (1.7%)
Difficulty starting urination, *n* (%)	5 (8.3%)	1 (1.7%)
Muscle cramps or stiffness, *n* (%)	5 (8.3%)	1 (1.7%)
Trouble catching breath or hyperventilation, *n* (%)	4 (6.7%)	1 (1.7%)
Chest pain, *n* (%)	4 (6.7%)	1 (1.7%)
Trouble concentrating, *n* (%)	3 (5.0%)	1 (1.7%)
Ringing in ears or trouble hearing, *n* (%)	3 (5.0%)	1 (1.7%)
Feeling strange or unreal, *n* (%)	2 (3.3%)	1 (1.7%)
Clenching of teeth at night, *n* (%)	2 (3.3%)	1 (1.7%)
Abnormal sensations, *n* (%)	0 (0.0%)	1 (1.7%)
Weakness or fatigue, *n* (%)	8 (13.3%)	0 (0.0%)
Increased appetite, *n* (%)	8 (13.3%)	0 (0.0%)
Feeling nervous or hyper, *n* (%)	7 (11.7%)	0 (0.0%)
Frequent need to urinate, *n* (%)	6 (10.0%)	0 (0.0%)
Trouble sitting still, *n* (%)	6 (10.0%)	0 (0.0%)
Unable to sit still, *n* (%)	6 (10.0%)	0 (0.0%)
Difficulty finding words, *n* (%)	5 (8.3%)	0 (0.0%)
Rapid or pounding heartbeat, *n* (%)	5 (8.3%)	0 (0.0%)
Feeling drowsy or sleepy, *n* (%)	5 (8.3%)	0 (0.0%)
Fluid retention or swelling, *n* (%)	4 (6.7%)	0 (0.0%)
Constipation, *n* (%)	4 (6.7%)	0 (0.0%)
Poor coordination or unsteadiness, *n* (%)	4 (6.7%)	0 (0.0%)
Blurred vision, *n* (%)	4 (6.7%)	0 (0.0%)
Dizziness or faintness, *n* (%)	4 (6.7%)	0 (0.0%)
Strange taste in mouth, *n* (%)	3 (5.0%)	0 (0.0%)
Dizziness when standing up, *n* (%)	3 (5.0%)	0 (0.0%)
Tremor or shakiness, *n* (%)	3 (5.0%)	0 (0.0%)
Muscle twitching or movements, *n* (%)	2 (3.3%)	0 (0.0%)
Diarrhea, *n* (%)	2 (3.3%)	0 (0.0%)
Bruising, *n* (%)	2 (3.3%)	0 (0.0%)
Hair thinning or loss, *n* (%)	1 (1.7%)	0 (0.0%)
Slurred speech, *n* (%)	1 (1.7%)	0 (0.0%)
Drooling or increased salivation, *n* (%)	0 (0.0%)	0 (0.0%)

*Note:* Numbers and percentages of patients reporting moderate‐to‐severe increases in specific symptoms on the Systematic Assessment for Treatment Emergent Effects (SAFTEE). Adverse events are listed based on severe increases in symptoms, with the most frequent severe increased symptoms at the top. Total sample includes patients with a baseline SAFTEE measurement and at least one follow‐up SAFTEE measurement (*N* = 60).

Four deaths were reported: three patients died by physician‐assisted death, and one patient died from pneumonia. None of the four deaths was considered by the physician or researcher to be causally related to oral esketamine maintenance treatment. All three patients who died by physician‐assisted death were treated with oral esketamine towards the end of life and reported that their depressive symptoms were somewhat more bearable due to esketamine treatment. No adverse events indicating esketamine (or ketamine) abuse, hepatotoxicity, or neurocognitive impairment were reported.

Of the 53 patients who discontinued maintenance treatment, three (5.7%; 3.2% of *N* = 93) did so because of adverse events, and nine (17.0%; 9.7% of *N* = 93) did so due to the combination of adverse events and insufficient sustained improvement (*N* = 8) or lack of insurance coverage of treatment (*N* = 1) (Figure 1). These adverse events were suspected esketamine‐related reactions based on the available data at the time of discontinuation. They included acute effects (e.g., dissociation and nausea) (*N* = 5), lower urinary tract symptoms (*N* = 4), and exacerbation of symptoms at or after treatment days (e.g., irritability, fatigue, and depressed mood) (*N* = 3). With regard to the urinary tract symptoms, two patients showed remission of these symptoms after the discontinuation of esketamine, suggesting that the symptoms were indeed related to esketamine treatment. One patient was found to have a ureteral stone, which was unlikely to be related to esketamine treatment, and one patient appeared to have urinary retention. In the latter case, symptoms were considered to be related to oral esketamine maintenance treatment by the physician at the time of discontinuation. However, as urinary tract symptom severity increased after the discontinuation of esketamine treatment, a causal relationship with esketamine treatment became less likely.

## 4. Discussion

This is the first study to describe the clinical outcomes during at‐home oral esketamine maintenance treatment in patients with TRD. Additionally, this is one of the larger studies to date to report on any form of ketamine maintenance treatment for depression, with a mean treatment duration of 20.8 months being among the longest reported in the literature [10–13].

In our uncontrolled cohort of 93 patients with severe TRD, we found no change at the group level in depression symptom severity between the maintenance treatment baseline and the last observation. These findings suggest that initial antidepressant effects were sustained during oral esketamine maintenance treatment and are consistent with previous group‐level results from studies on IN esketamine [11, 45] and IV ketamine [46] maintenance treatment. However, examination of individual‐level data showed that symptom severity varied over the course of maintenance treatment in the majority of patients, including trajectories of late‐onset response, intermittent response, and (partial) relapse without a subsequent response. Given the observational design of this study, no causal inference can be made regarding the relationship between oral esketamine maintenance treatment and any of these trajectories. In line with this, the latter two trajectories should not be interpreted as oral esketamine maintenance treatment necessarily being completely ineffective in sustaining the initial antidepressant effect of oral esketamine. First, we did not account for potential third factors, such as life events and esketamine tapering attempts, which could have affected depressive symptom severity. Additionally, given the severity of treatment resistance in this population—with an average episode duration of over 6 years, eight failed antidepressant trials, insufficient response to ECT in 65.4%, a lifetime history of five psychotherapies, and psychiatric comorbidity in 74.1%—one might argue that the fact that 23.1% of initial responders met sustained response criteria and 53.8% of initial responders met response criteria intermittently is clinically significant. This is supported by the MCID data, which show that a majority of patients met initial MCID criteria, and of those, the majority also met sustained MCID criteria.

Although several previous ketamine and esketamine maintenance studies have reported higher sustained response rates [10, 12], similar rates to ours were found in studies on maintenance treatment in patients with severe TRD, as well as in studies with longer follow‐up periods [10]. This may be related to the fact that patients with TRD have a higher risk of relapse and to the general decline in depression recovery rates over time [5, 47]. Still, as relapse frequently occurred, regardless of the reason, we need to optimize ketamine maintenance treatment. Strategies for optimization may lie in a symptom‐driven approach, where adjustments of the dosing based on symptom severity would allow the ability to potentially interrupt early signs of relapse as well as prevent overtreatment of patients who demonstrate stable remission. Another potential strategy is the addition of carefully timed (digital) psychological interventions [48]. In addition, future head‐to‐head studies are warranted to study the relative effectiveness of oral esketamine maintenance treatment versus other routes of ketamine and esketamine administration.

Of the 53 patients who discontinued maintenance treatment, 41.5% did so (partly) because of insufficient sustained improvement. Maintenance baseline scores on the HDRS_17_ (17.06) and IDS‐SR (42.69) were notably higher in this subgroup compared to the subgroup still receiving maintenance treatment at the time of the database lock (10.75 and 29.88, respectively). This suggests that patients who exhibit severe symptoms at the end of induction treatment may be at greater risk of discontinuing maintenance treatment due to insufficient sustained improvement at some point during treatment. However, we believe that initial nonresponse or persistent severe depressive symptoms at the end of induction treatment would not necessarily provide an irrefutable reason for exclusion from maintenance treatment, as was the case in most larger maintenance studies [11, 45, 49]. We propose that in real‐world practice, the decision to (dis)continue treatment should be a collaborative choice between the patient and the physician, taking into account several clinical aspects, including the perceived effectiveness of treatment, its clinical value and duration (i.e., whether it provides more than just an acute positive effect), side effects, and the availability (or lack) of treatment alternatives. In addition, since almost half of the initial nonresponders met response criteria at a later time during maintenance treatment, it seems important not to abandon efforts too soon.

The fact that some patients continued esketamine treatment despite not meeting criteria of initial response or even MCID suggests that there may be a benefit that cannot be captured by depression‐specific instruments such as the HDRS_17_ and IDS‐SR. A previous study on IV ketamine maintenance treatment in patients with high levels of TRD found similar results: while only 18.3% of patients met response criteria, almost 50% transitioned to maintenance treatment [50]. Questionnaires on quality of life or qualitative interviews may be more suitable to capture the full extent of improvement [51]—especially in this population with severe TRD, where treatment protocols have been exhausted and no longer offer alternatives.

In addition to the key issue of the trajectory of depressive symptom severity during maintenance treatment, there are concerns regarding its safety profile. Potential harmful consequences of prolonged ketamine use include addiction, ulcerative cystitis, hepatoxicity, and neurocognitive impairment [14–17]. Adverse events related to seeking, overdosing, or abuse of esketamine (or ketamine) were not reported. However, (mild symptoms of) dependency may have gone undetected. To monitor addiction in a more structured way, we have recently introduced the ketamine side effect tool (KSET) [52], which includes questions regarding craving and seeking and using non‐prescribed ketamine or esketamine.

Four deaths were reported. None was considered causally related to oral esketamine maintenance treatment. However, this mortality rate exceeds that of the general Dutch population. The increased mortality risk associated with depressive disorders and TRD in particular may partly explain this finding. Moreover, maintenance treatment was offered only after established treatment options had been exhausted or when there were well‐founded reasons not to pursue them. Therefore, this cohort included many patients with limited perspective, and several patients had already entered, or were in the process of entering, a euthanasia assessment process when they initiated esketamine as a last‐resort treatment. In patients who did not meet sustained response to esketamine, continuation of this process probably contributed to the observed mortality rate.

Four patients discontinued treatment due to urinary tract symptoms, and in three of those symptoms were possibly (*N* = 1) or likely (*N* = 2) related to esketamine treatment. Urinary tract symptoms resolved in two of the patients after the discontinuation of esketamine, but in one patient, symptoms increased thereafter. Although the course of the symptoms argues against it, a relationship with esketamine treatment cannot be ruled out completely, and this should be taken seriously. Moderate and severe increases in symptoms potentially related to ulcerative cystitis were reported by some patients via the SAFTEE. However, rates were comparable to those observed in a similar cohort of patients who did not receive esketamine [32]. Overall, in line with previous research [10, 53], serious urinary tract problems related to ketamine or esketamine treatment seem uncommon. However, we must remain vigilant as current data are still limited, follow‐up time is generally short, and, if left unaddressed, mild and recoverable urinary tract problems could progress to severe and incurable problems.

With regard to hepatotoxicity and neurocognitive impairment, physicians administering esketamine treatment were advised to monitor liver enzyme levels and cognitive function every 6 months. Although we do not evaluate all of these data ourselves, no issues related to hepatotoxicity or neurocognitive impairment were spontaneously reported by physicians nor by patients, during monitoring assessments or through the open‐ended inquiry of the SAFTEE.

Ketamine and esketamine induction treatment is known to be relatively well‐tolerated [54]. Similarly, we found oral esketamine maintenance treatment to be well‐tolerated, with only three patients discontinuing treatment entirely due to adverse events. This finding is consistent with previous studies on maintenance treatment [10, 11, 18]. The adverse events reported were also in line with those previously associated with ketamine and esketamine treatment [39, 54]. However, one adverse event merits comment. We found that symptoms of sexual dysfunction were the most common severely increased adverse events. Sexual dysfunction has been described in preclinical studies and in individuals using nonmedical ketamine [55–58]. However, to our knowledge, sexual dysfunction has not previously been reported in association with ketamine or esketamine treatment in depression before. This could be explained by the fact that assessment of sexual dysfunction is uncommon in previous studies [59], combined with the finding that relying on spontaneous report leads to underestimation of such symptoms [60]. An alternative hypothesis is that factors other than esketamine caused the increase in sexual dysfunction, e.g., depression relapse. Although not formally tested, based on the examination of the data, this explanation could be valid in at least some of the patients. Another possible explanation is that sexual dysfunction, which is itself a common symptom of depression, becomes more burdensome when other depressive symptoms improve. It also remains unclear whether greater exposure to esketamine’s metabolites following oral administration due to extensive first‐pass metabolism contributes to this finding. Given these considerations, we consider this observation to represent a hypothesis‐generating safety signal, and we would argue for routine, standardized assessment of sexual functioning in ketamine and esketamine studies to further investigate a potential relationship.

Oral administration of esketamine offers the possibility of at‐home treatment, having great feasibility, accessibility, and scalability potential. Although several experts have argued against this, mainly because of acute effects (e.g., hypertension and dissociation) and misuse potential [61], others suggest that at‐home treatment may be reasonable for a select group of patients [62]. Our findings suggest that at‐home treatment can be safe in patients with TRD. This is in line with recent findings on at‐home ketamine treatment for depression [63–65] and previous findings in the setting of chronic pain [29]. Based on these findings, at‐home treatment may be a reasonable alternative to in‐hospital treatment but only under the right conditions [58, 66].

This study has several limitations, including its open‐label design and the absence of a control group. In addition, in studies on psychedelics, including esketamine, expectancy bias is considered a major problem [67], potentially leading to an overestimation of the treatment effect. Third, our study is at risk for omitted variable bias, as relevant factors such as life events, changes in concomitant treatment, and (protocol‐driven) dose adjustments in case of depression relapse or attempts to taper esketamine treatment were not included in our analyses. Although the inclusion of these variables would have been of considerable interest, this was not feasible due to the absence of these data for most patients. Fourthly, our study is susceptible to selection bias as eligibility for maintenance treatment was based on subjective, qualitative criteria. To facilitate assessment of this potential bias, the clinical characteristics of patients who did not enter the maintenance phase are provided in the Supporting Information. In addition, we encountered a significant amount of missing data, which may have been nonrandom. We attempted to mitigate the impact of missing data by reporting both clinician‐rated and self‐rated questionnaires. In addition, we used as much available data as possible through individual‐level data analysis and limited the effect of missing interim data by the use of paired‐samples *t*‐tests. However, the latter analysis required the use of the last‐observation‐carried‐forward, which also has important limitations. Alternative missing‐data approached were explored but were not considered adequate given the high rates of missing data—likely not at random. Another limitation is the possibility that some adverse events went undetected, given that liver enzyme levels and data on cognitive functioning were not part of our dataset, and we had to rely on patient report. Our results should therefore be interpreted with these limitations in mind.

## 5. Conclusions

In conclusion, the findings from this real‐world cohort study suggest that at‐home oral esketamine maintenance treatment may be associated with sustained symptom improvement in patients with severe TRD. However, causal inferences cannot be drawn from this observational study, and the findings should be interpreted in light of several limitations. In addition, symptom severity varied over the course of maintenance treatment in the majority of patients, indicating that this relapse prevention strategy requires further optimization. Although some potential adverse events require monitoring and further examination, oral esketamine was overall safe and well‐ tolerated. Future head‐to‐head studies are warranted to study the relative effectiveness, safety, and tolerability of oral esketamine versus other routes of administration.

## Author Contributions

Sanne Y. Smith‐Apeldoorn wrote the treatment protocol with input from Jolien K. E. Veraart, Jeanine Kamphuis, and Robert A. Schoevers. Sanne Y. Smith‐Apeldoorn, Jolien K. E. Veraart, Jeanine Kamphuis, Jan Spijker, Annemarie Van Der Meij, and Robert A. Schoevers were responsible for the treatment and data collection. Sanne Y. Smith‐Apeldoorn conducted the statistical analysis and drafted the original manuscript. All authors contributed to the interpretation of the data. All authors contributed to the redrafts.

## Funding

This research received no specific grant from any funding agency in the public, commercial, or not‐for‐profit sectors.

## Disclosure

All authors had full access to all the data and had final responsibility for the decision to submit the article for publication.

## Conflicts of Interest

Jolien K. E. Veraart, Jeanine Kamphuis, and Jan Spijker received a speakers fee from Janssen Pharmaceuticals, outside the submitted work. Robert A. Schoevers received research funding for two randomized clinical trials with generic oral esketamine from the Netherlands Organisation for Health Research and Development and the National Health Care Institute, a speaker fee and investigator‐initiated research grant from Janssen Pharmaceuticals, and consultancy fees from GH Research, Beckley PsyTech, and QPS, outside the submitted work. The other authors declare no conflicts of interest.

## Supporting Information

Additional supporting information can be found online in the Supporting Information section.

## Supporting information


**Supporting Information** The Supporting Information includes clinical characteristics of the patients who did not enter the maintenance treatment program after induction treatment. Table 1: Clinical characteristics of patients with a treatment‐resistant unipolar depressive disorder who did not enter the maintenance treatment program after oral esketamine induction treatment. As an addition to Figures 2 and 3 the Supporting Information includes details on HDRS_17_ total scores and IDS‐SR total scores over the course of maintenance treatment. Table 2: Mean Hamilton Depression Rating Scale total score. Table 3: Mean Inventory of Depressive Symptomatology—Self Report total score.

## Data Availability

The data that support the findings of this study are available upon request from the corresponding author. The data are not publicly available due to privacy or ethical restrictions.
