# Supplementary material for: Oral Esketamine Maintenance Treatment for Treatment‐Resistant Depression: Clinical Outcomes During an Off‐Label Treatment Program
Source: Depress Anxiety. 2026 Oct 29;2026:8320239. doi: 10.1155/da/8320239 (PMC13719807; doi:10.1155/da/8320239)
Supplement: Supplementary file 1 [file DA-2026-8320239-s001.docx]

**Supplementary Information**

**Supplementary Table 1: Clinical characteristics of patients with a treatment-resistant unipolar depressive disorder who did not enter the maintenance treatment program after oral esketamine induction treatment.**

| **Characteristic** | **No maintenance treatment**  **N = 100** |
| --- | --- |
| Duration current episode at baseline, months, mean (SD)^a^ | 70.1 (84.24) |
| No of depressive episodes, mean (SD)^a^ | 6.5 (13.23) |
| No of antidepressants lifetime, mean (SD)^b^ | 8.1 (3.08) |
| History of ECT, N (%)^c^ | 42 (56.8) |
| History of psychotherapy (> 12 sessions), N (%)^d^ | 47 (71.2) |
| HDRS_17_ total score at baseline, mean (SD)^e^ | 22.08 (4.904) |
| HDRS_17_ total score change between baseline and end of induction treatment, mean (SD)^f^ | 2.72 (5.324) |
| AE burden (SAFTEE) at end of induction treatment, mean (SD)^g^ | 1.56 (0.990) |

^a^ N = 77; ^b^ N = 73; ^c^ N = 74; ^d^ N = 66; ^e^ N = 71; ^f^ N = 60; ^g^ N = 45, in maintenance treatment sample AE burden at the end of induction treatment was 1.49 (1.044); AE = adverse events; ECT = electroconvulsive therapy; HDRS_17 =_ Hamilton Depression Rating Scale; SAFTEE = Systematic Assessment for Treatment Emergent Effects.

**Supplementary table 2: Mean Hamilton Depression Rating Scale Total Score.**

| **Month** | **Number of patients^a^** | **Mean HDRS_17_** | **Standard error** |
| --- | --- | --- | --- |
| -1 | 82 | 20.59 | .71 |
| 0 | 75 | 12.43 | .73 |
| 1 | 26 | 11.81 | 1.18 |
| 2 | 34 | 11.71 | 1.00 |
| 3 | 29 | 12.34 | 1.29 |
| 4 | 35 | 14.40 | 1.31 |
| 5 | 30 | 13.07 | 1.49 |
| 6 | 23 | 11.00 | 1.17 |
| 7 | 28 | 12.75 | 1.30 |
| 8 | 23 | 12.26 | 1.30 |
| 9 | 26 | 13.88 | 1.42 |
| 10 | 21 | 12.24 | 1.61 |
| 11 | 25 | 13.32 | 1.45 |
| 12 | 18 | 12.22 | 1.53 |
| 13 | 17 | 12.71 | 1.36 |
| 14 | 21 | 13.14 | 1.57 |
| 15 | 18 | 12.17 | 1.42 |
| 16 | 17 | 11.00 | 1.72 |
| 17 | 14 | 11.64 | 1.96 |
| 18 | 17 | 12.82 | 1.84 |
| 19 | 15 | 11.00 | 1.81 |
| 20 | 13 | 8.69 | 2.16 |
| 21 | 10 | 9.90 | 2.54 |
| 22 | 10 | 10.80 | 2.54 |
| 23 | 7 | 8.14 | 2.39 |
| 24 | 11 | 10.27 | 1.57 |

Mean Hamilton Depression Rating Scale (HDRS_17_) Total Score at baseline of the induction phase (month = -1), at baseline of the maintenance phase (month = 0), and over the course of maintenance treatment up to 24 months. ^a^ Number of patients of whom the HDRS_17_ Total Score for that month was available.

**Supplementary table 3: Mean Inventory of Depressive Symptomatology – Self Report Total Score.**

| **Month** | **Number of patients^a^** | **Mean IDS-SR** | **Standard error** |
| --- | --- | --- | --- |
| -1 | 73 | 46.08 | 1.40 |
| 0 | 71 | 33.73 | 1.72 |
| 1 | 35 | 31.66 | 2.77 |
| 2 | 38 | 30.45 | 2.46 |
| 3 | 41 | 31.34 | 2.17 |
| 4 | 45 | 31.44 | 2.31 |
| 5 | 41 | 31.61 | 2.20 |
| 6 | 41 | 33.00 | 2.25 |
| 7 | 43 | 31.58 | 2.48 |
| 8 | 44 | 30.39 | 2.19 |
| 9 | 44 | 31.52 | 2.20 |
| 10 | 43 | 31.91 | 2.18 |
| 11 | 40 | 32.02 | 2.24 |
| 12 | 38 | 31.45 | 2.68 |
| 13 | 40 | 28.70 | 2.45 |
| 14 | 38 | 29.92 | 2.45 |
| 15 | 31 | 31.26 | 2.88 |
| 16 | 37 | 27.62 | 2.34 |
| 17 | 41 | 28.07 | 2.42 |
| 18 | 34 | 30.97 | 2.60 |
| 19 | 37 | 30.97 | 2.39 |
| 20 | 33 | 28.97 | 2.45 |
| 21 | 32 | 28.09 | 2.78 |
| 22 | 29 | 27.86 | 2.65 |
| 23 | 29 | 28.17 | 2.70 |
| 24 | 29 | 29.14 | 2.89 |

Mean Inventory of Depressive Symptomatology – Self Report (IDS-SR) Total Score at baseline of the induction phase (month = -1), at baseline of the maintenance phase (month = 0), and over the course of maintenance treatment up to 24 months. ^a^ Number of patients of whom the IDS-SR Total Score for that month was available.
